# Case Report: Infertility in bitches at a German Shepherd breeding center and nutritional management

**DOI:** 10.3389/fvets.2026.1801445

**Published:** 2026-06-02

**Authors:** Livio Penazzi, Maria Carmela Pisu, Mario Agrillo, Liviana Prola

**Affiliations:** 1 Department of Veterinary Sciences, University of Turin, Grugliasco, Italy; 2 Centro di Referenza Veterinario (VRC), Turin, Italy; 3 Pet Food Project, Rome, Italy

**Keywords:** anoestrus, bioavailability, canine, deficiency, dog, nutrition

## Abstract

**Background:**

Inadequate nutrition of the dams, even if recognized as a possible cause of infertility, it is usually underestimated during the common diagnostical procedures (i.e., clinical examination, vaginal cytology, ultrasound, blood and urine analysis). Although the diet may meet adult maintenance requirements, several nutrients can be suboptimal during the pre-gestation period and may influence reproductive performance. This case report describes the use of an oral supplement to increase fertility in a German Sheperd breeding center experiencing prolonged secondary anoestrus.

**Case presentation:**

Fifteen German Shepherd bitches with secondary anoestrus (>12 months) underwent standard reproductive workup (vaginal cytology, hormone assays, ultrasound, and semen testing of sires). Abdominal ultrasound of the bitches did not present any signs of cystic endometrial hyperplasia (CEH). Similarly, metabolic disorders (hypothyroidism and Cushing’s disease) were ruled out. It was hypothesized that the kennel diet might have provided suboptimal bioavailability of nutrients; as such the nutritional plan was reviewed, and a multi-component “fertility mix” (liquid and powder phases dosed daily at 0.8 g/kg^0.75^ and 0.4 g/kg^0.75^, respectively) was added to the usual complete maintenance ration.

**Results:**

Within 6 months of supplementation, 12/15 bitches resumed cyclicity and were confirmed pregnant or gave birth; whereas three underweight bitches with chronic enteropathies did not resume cyclicity.

**Conclusion and limitations:**

The diagnostic approach to infertility is complex and involves both male and female factors, while dietary adequacy is often overlooked. Nutrients such as eicosapentaenoic acid (EPA), docosahexaenoic acid (DHA), arachidonic acid, folic acid, vitamin E, selenium, and zinc may influence ovarian steroidogenesis and antioxidant systems, and maintenance diets may not always be optimal for bitches intended for reproduction. These observations are hypothesis-generating; baseline and follow-up nutrient biomarkers were not obtained and no untreated control group was available, precluding causal inference.

## Background

Dogs have unique reproductive features compared to other domestic animals (1). They are non-seasonal monoestrus mammals with one breeding cycle in a season for most of the breeds. Each canine cycle is characterized by four different phases: proestrus (5–20 days), oestrus (5–15 days), dioestrus (50–80 days), and anoestrus (80–240 days) (2). Canine anoestrus is characterized by the absence of ovarian activity and involves a phase of endometrial repair after progesterone declines below 1–2 ng/mL (3). Serum oestradiol and basal luteinizing hormone (LH) are both low during this phase (5–10 pg./mL and <1–2 ng/mL respectively), while follicle-stimulating hormone (FSH) is high (50–400 ng/mL) (2).

Prolonged interoestrus intervals can be one of the factors affecting infertility in dams (4); especially in those breeds, such as German Shepherd dogs, where the interoestrus cycle is known to be relatively short (4–5 months) (5). In fact, secondary anoestrous can be defined as the loss of cyclicity in those bitches which have had previous oestrus cycles (6). Several authors have investigated causes of infertility and pregnancy loss, in particular pointing out (when the breeding management problems were ruled out): systemic disease (in conjunction with clinical signs of excessive or abnormal vaginal discharge, vaginal mucosal inflammation, peripheral leucocytosis), vaginal infection (e.g., *brucella canis*), endocrinologic dysfunction (hypothyroidism or Cushing’s disease) or ovarian neoplasia (4, 5, 7–10). However, limited data are available on the effect of nutrition on the canine reproductive cycle and fertility; while in human medicine the relation between diet and pregnancy success rate has been well established (11, 12). According to Ogoshi et al. (13), the dam should be well fed and with an adequate Body Condition Score (BCS) before the beginning of the gestation, but the authors do not mention any specifics about the different nutrients requirements. While obesity is a well-known factor associated with infertility, silent heat, prolonged interoestrous intervals and anoestrus in dogs (14), little attention has been given to the nutritional needs of underweight dogs. Lawler et al. (15) reported that undernutrition (receiving 75% of the food quantity compared to maintenance levels), even if may not affect directly the oestrus cycle, may hinder the bitches ability to become pregnant. A poor body condition score has been associated to metabolic disturbances mainly in gestating and lactating bitches, such as gestational ketosis, impaired endocrine equilibrium, impaired placentation, increased neonatal death rates (16, 17). Nevertheless, the effect of underweight status and malnutrition has been correlated with hypofertility in humans (18). Restoration of body weight and micronutrients deficiencies (such as vitamin B6, vitamin B12, folic acid, vitamin E, magnesium, selenium, iron and zinc) has been shown to improve women fertility (19). In dams it was observed that the supplementation of zinc, copper, manganese, folate, L-Arginine, and an adequate linoleic: α-linolenic acid ratio (LA: ALA) has been associated with improved reproductive efficiency in some studies; however, it is difficult to correlate the individual effect of each of these nutrients to their respective genital function stimulation (20, 21). Nonetheless, mechanisms of action have been hypothesized of some of these nutrients in the reproductive performance. For example, it was assessed that folate and homocysteine are present in the follicular fluid in humans, proportionally to the circulating levels (22). Therefore, due to its role in the synthesis, repair and methylation of DNA, a deficiency in folic acid could compromise oocyte competence and development as well as early embryogenesis (23). Similarly, zinc deficiency appears to cause abnormal or failed development of germ cells in both sexes (24). In particular, studies conducted on mice showed an impaired follicle rupture during ovulation (25, 26), which is allegedly due to the decrease activity of zinc-dependent enzymes, such as metalloproteinases, which are essential for the follicular wall degradation (27). In animal models, the supplementation of omega-3 fatty acids [α-linolenic acid (ALA), eicosapentaenoic acid (EPA), docosahexaenoic acid (DHA)] has been correlated to a modification in the prostaglandin biosynthetic pathways, improving the folliculogenesis, oocyte maturation, ovulation, implantation and prolongation of the female reproductive lifespan (28). In rats, selenium deficiency was associated with ovarian degeneration and follicle atresia (29), with higher infertility occurrence, similar to human studies (30), probably due to the relation between Selenium (Se) status and the Se-dependent glutathione peroxidase activity (31).

Therefore, this case report aimed to explore whether the unsuccessful pregnancy rate of the bitches in a breeding center could be related to nutritional deficiencies after eliminating other possible clinical causes of infertility.

## Case presentation

A private family-owned breeding center with 15 active German Shepherd dams, with median age of 3 years old (Interquartile Range [IQR] 2.5–4), average body weight of 27.1 (±2.52) kg and median BCS of 4 out of 9 (IQR 4–5) according to Laflamme (32), requested a nutritional consult after no one of the dams was able to return to oestrus after 12 months or more from the previous one. The dams were presented at the nutritional consultation sector of the Department of Veterinary Sciences, University of Turin. All procedures were conducted in accordance with institutional guidelines for clinical veterinary practice, and the owner of the breeding kennel provided written informed consent for all diagnostic and nutritional interventions performed during the clinical evaluation. Formal ethical committee approval was not sought because the dogs were managed within the context of routine clinical care and no experimental procedures outside standard veterinary practice were performed, and no animal was subjected to invasive sampling beyond those required for diagnostic purposes. Institutional ethical committee approval was not obtained for publication of this case report because the intervention was undertaken as part of routine clinical management. No major changes in housing conditions or kennel management were reported during the observation period.

**Figure 1:**
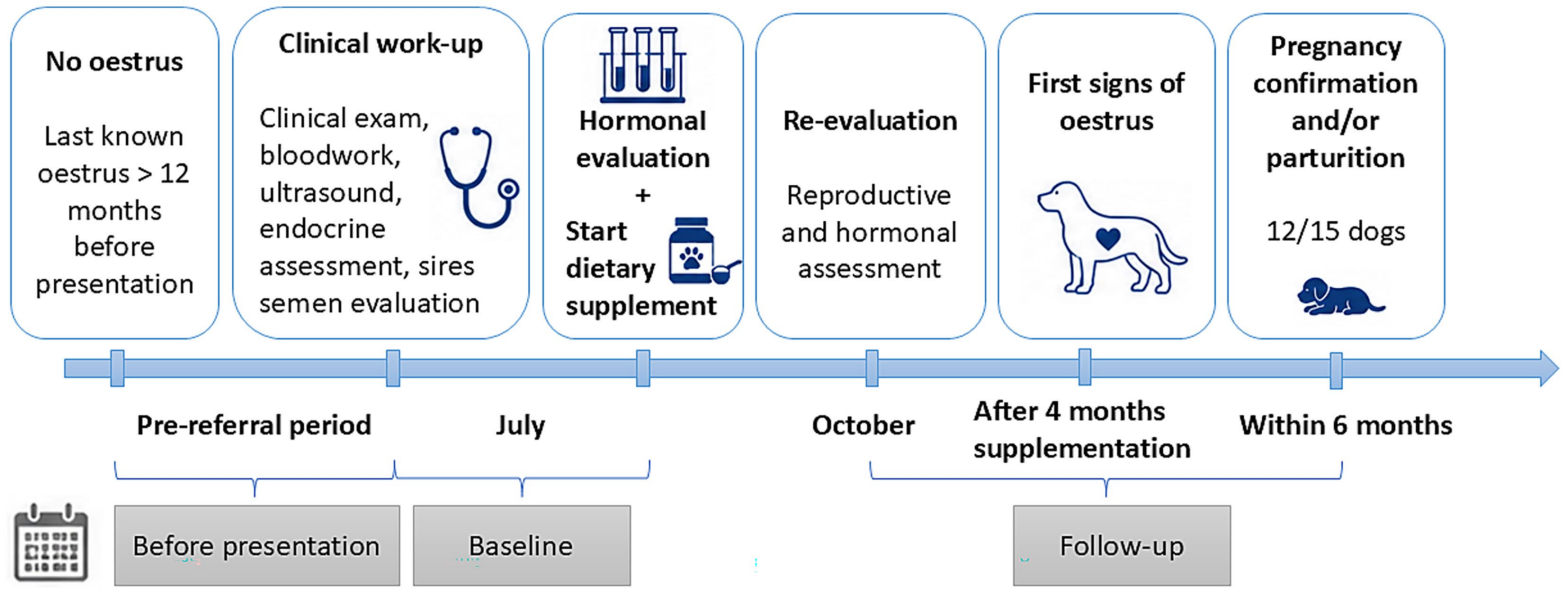
Timeline of the main diagnostic, nutritional, and reproductive events.

The dogs were previously evaluated by the referring veterinarians of the breeding center for blood analysis: Complete Blood Count [CBC] and biochemistry (total protein, albumin, globulin, CK, total bilirubin AST, ALT, ALP, GGT, cholesterol, triglycerides, amylase, lipase, BUN, creatinine, glucose, calcium, phosphorus, magnesium, sodium, potassium, chloride and C-reactive protein), but no variations of the parameters were found. During the previous year regular vaginal cytology evaluations of the bitches were persistently consistent with dioestrus or anoestrus. Vaginal cytology was repeated before supplementation and during follow-up to monitor resumption of cyclicity. Baseline hormonal testing (LH, progesterone and oestradiol) was performed in July, before supplementation was introduced, and repeated in October during follow-up (Figure 1). Luteinizing hormone was evaluated as part of the reproductive assessment to investigate possible ovarian dysfunction, but all the bitches showed LH levels below 1 ng/mL, making this diagnosis less likely. On July progesterone levels were below 1 ng/mL for all the tested dogs with mean 0.2 ± 0.1 ng/mL and oestradiol was consistently < 10 pg./mL, as all the dogs resulted to be in anoestrus.

Abdominal ultrasound did not present in any of the bitches signs of cystic endometrial hyperplasia (CEH). Similarly, the dogs were tested for metabolic disorders (hypothyroidism and Cushing’s disease), but resulted negative. No hormonal pharmacological therapy was performed in any of the dogs. Three of the dogs had enteropathies which etiology was still to be assessed and presented the lowest BCS (3) among the bitches. The 3 dogs with gastroenteric symptoms were also tested for Giardia but resulted negative. Aside from these 3 dogs all the other animals were clinically healthy. The males used for reproductive purposes were also tested for seminal fluid characteristics (spermatozoa morphology, motility, sperm count) and all 3 resulted normal with a high insemination rate, excluding them from a possible cause of missed pregnancy rate.

The dogs were given (ad libitum) a commercial dry diet containing the following ingredients (as declared on the label): corn, chicken and turkey dehydrated meat, rice (12%), fresh chicken and turkey (10%), lard, hydrolysed chicken protein, beet pulp (2.3%), linseed, dehydrated fish meal, sodium chloride, pea fiber, brewer’s yeast, calcium carbonate, green mussel extract (*Perna canaliculus*, 0.01%), spirulina (*Arthrospira platensis*, 0.005%), dried apple (0.0025%). The nutritional composition as well as the ration additives are reported in Table 1. Dry food was continuously available to the dogs, and individual daily food intake could not be reliably quantified, as feeding was conducted under routine breeding-center conditions rather than within a controlled experimental setting.

**Table 1:** Nutritional composition and additives of the diet administered to the bitches.

Nutrient and chemical composition	Unit	As fed
Dry matter	%	92.0
Organic matter	%	86.2
Crude protein	%	24.0
Ether extract	%	14.0
Crude fiber	%	2.3
Ash	%	5.8
Calcium	%	0.95
Phosphorus	%	0.75
Metabolizable energy	Kcal/100 g	385.1
Metabolizable energy	MJ/100 g	1.61

Additives	Unit	As fed
Vitamin A	IU/kg	26,000
Vitamin D3	IU/kg	1,300
Vitamin E	mg/kg	355
Copper (copper sulfate pentahydrate)	mg/kg	50
Iron (Ferrous carbonate)	mg/kg	350
Iodine (calcium iodate, anhydrous)	mg/kg	25
Selenium (sodium selenite)	mg/kg	20
Zinc (zinc oxide)	mg/kg	160
Manganese (manganous oxide)	mg/kg	57
Choline chloride	mg/kg	1,200

After assessment of the dietary management proposed by the breeder, it was decided to supplement the diet with a premix (“fertility mix”), formulated by the authors for this purpose, composed of both a liquid and powder phase as described on Table 2. Linseed oil was included as a source of α-linolenic acid to contribute to the overall polyunsaturated fatty acid profile of the supplement. Supplement dosing was calculated as 0.8 g/kg^0.75^ for the liquid phase and 0.4 g/kg^0.75^ for the powder phase. For a bitch weighing 27 kg, this corresponded to approximately 9.5 g/day of the liquid phase and 4.7 g/day of the powder phase; the corresponding estimated daily nutrient contribution is reported in Table 3. Supplementation was administered daily, with both the powder and liquid phases mixed into a small portion of the usual ration. This portion was offered first to ensure full consumption of the supplement, after which the remainder of the daily ration was provided. Supplement acceptance was reported as excellent by the breeder with the dogs always accepting the ration mixed with the supplement.

**Table 2:** Ingredient and nutritional composition of the “fertility mix.”

Ingredient	Unit	As fed
Liquid phase		
Sunflower oil	%	57.35
Salmon oil	%	37.43
Linseed oil	%	5.21
Powder phase additives		
Dehydrated pork meat	%	97.00
Zinc (zinc glycinate)	%	2.53
α-Tocopherol	%	0.25
Arachidonic acid	%	0.22
Folic acid	%	0.002

Nutritional composition	Unit	As fed
Liquid phase		
Dry matter	%	99.9
Organic matter	%	99.9
Crude protein	%	0
Ether extract	%	99.9
Crude fiber	%	0
Ash	%	0
Metabolizable energy	Kcal/100 g	933.0
Linoleic acid	%	31.6
α-Linolenic acid	%	0.38
Arachidonic acid	%	0.66
EPA	%	2.25
DHA	%	2.25
Vitamin E	mg/100 g	21.6
Powder phase additives		
Dry matter	%	95.46
Organic matter	%	94.14
Crude protein	%	49.89
Ether extract	%	13.61
Crude fiber	%	8.62
Ash	%	1.32
Metabolizable energy	Kcal/100 g	372.8
Zinc	mg/100 g	657.93
Arachidonic acid	%	0.62
Folates	mg/100 g	2.11
Vitamin E	mg/100 g	125

**Table 3:** Estimated daily nutrient contribution of the “fertility mix” for a 27.0 kg bitch.

Parameter	Liquid phase	Powder phase	Total estimated daily intake from supplement
Dose (g/day)	9.5	4.7	14.2
Metabolizable energy (Kcal/day)	88.6	17.5	106.1
Linoleic acid (g/day)	3.0	0.0	3.0
α-Linolenic acid (g/day)	0.036	0.0	0.036
Arachidonic acid (g/day)	0.063	0.029	0.092
EPA (g/day)	0.214	0.0	0.214
DHA (g/day)	0.214	0.0	0.214
Zinc (mg/day)	0.0	30.9	30.9
Folates (mg/day)	0.0	0.1	0.1
Vitamin E (mg/day)	2.05	5.88	7.93

Resumption of cyclicity was defined based on the appearance of clinical signs consistent with oestrus, supported by hormonal findings and vaginal cytology when available. After approximately 4 months of supplementation with the “fertility mix,” 12 of 15 bitches were observed to show signs of oestrus and 6 were already confirmed pregnant by ultrasound. Mean body weight at follow-up before pregnancy confirmation was 28.2 (±2.52) kg and median BCS of 5 out of 9 (IQR 5–5). The levels of progesterone were also in range with the ovulation and post-ovulation period with a mean of 6.4 ± 3.4 ng/mL. Within 6 months of initiating supplementation, the 12 dogs that resumed cycling were confirmed pregnant or gave birth. The only 3 dams which did not show oestrus were the underweight dogs with associated enteropathies which were still under treatment at the last known clinical check.

## Discussion

Nutritional assessment is not always systematically incorporated into the diagnostic work-up of canine infertility, despite increasing recognition that dietary composition may influence reproductive physiology. At the same time, the relevance of nutrition during the reproductive phase is reflected by the availability of commercial complete diets and supplements specifically intended for breeding animals, suggesting a growing practical interest in this field. Several articles cite different causes of infertility but no one focuses on nutrition or even mention it during the clinical approach (4, 5, 7–9). Although minimum nutrient requirements for dogs are defined primarily for gestation and lactation (33, 34), it is possible that bitches intended for breeding may have needs that differ from those met by standard maintenance diets. Moreover, these requirements ensure adequacy, which not necessarily means optimal nutrient provision for reproductive performance. In fact, a proper nutritional management should be achieved before mating and continued during the gestation and lactation period (20). In addition, essential fatty acids, vitamins and minerals are known to participate in ovarian steroidogenesis, uterine protein synthesis, placentation and fetal development (14, 35).

It is important to notice that not only the level of nutrients but also the chemical form of the diet constituents is essential to ensure good digestibility and absorption. In the present case report, even if the diet could be considered complete from a nutritional perspective, it is possible that the bioavailability of some nutrients was lower than expected. For example, inorganic zinc sources such as zinc oxide have been reported to have lower bioavailability compared with some organic complexes such as zinc glycinate (21). In addition to ingredient composition and nutrient form, storage conditions and shelf life may also affect the effective nutrient content of commercial diets, particularly for more labile compounds. In this case report, direct laboratory analysis of the commercial diet was not performed, and nutrient adequacy was therefore inferred from the manufacturer’s label declaration rather than from measured composition, partly because of budget constraints.

Polyunsaturated fatty acids (PUFAs) have been associated with multiple aspects of reproductive function, including follicular development and ovulation (20). Indeed, ovarian steroidogenesis can be affected by prostaglandin production, which in turn can be stimulated by increasing the essential fatty acids content (36). Females of several mammalian species can alter the number and size of ovarian follicles, the ovulation rate and progesterone production by the corpus luteum when fed diets with different PUFAs content (36). Nonetheless, readily available EPA and DHA sources influence both sperm motility and fertility (37), and the lack of these nutrients in the diet may have contributed to impaired reproductive performance as well. Although EPA and DHA are acknowledged in nutritional recommendations for canine reproduction, mainly in relation to gestation and early lactation, their potential contribution during the pre-gestational phase and to the regulation of cyclicity remains to be clarified in bitches.

Although individual daily food intake was not available, the supplement provided a measurable additional intake of selected nutrients and energy on the basis of the prescribed dose. Estimated daily contributions are reported in Table 3 and may help inform the design of future prospective studies. However, accurate comparison of nutrient intake before and after supplementation could not be performed, owing to the absence of measured individual food intake and of a full analytical characterization of the base diet.

Certain micronutrients may play a major role especially in during different phases of life. In particular, the importance of the folic acid supplementation has already been highlighted in the canine species, and especially in the bitch (20, 38). Similarly, the antioxidant system of the dams is crucial to preserve cells from free radicals damage. Supplementation with selenium and vitamin E showed improved fertility in male dogs (37, 39, 40), and since the female reproductive tract harbors various antioxidant enzymes (41), it is possible that supplementation with these nutrients could influence reproductive physiology, although this remains speculative in the present case report. Cholesterol may also be relevant in this context, given its central role as a precursor for steroid hormone synthesis; however, serum cholesterol values were within the laboratory reference range in the present case series.

Taken together, these observations suggest that nutritional factors merit consideration during the clinical evaluation of reproductive disorders, particularly when common endocrine or infectious causes have been excluded. However, the present case report can only describe a positive association between dietary supplementation and reproductive outcomes but cannot establish mechanistic links or treatment efficacy. The findings should therefore be interpreted as preliminary and exploratory, supporting the need for controlled prospective investigations integrating detailed dietary assessment, biochemical markers of nutrient status, and standardized reproductive evaluations.

## Conclusion

In this case report, introduction of a multi-component nutritional supplement was associated with resumption of oestrus and subsequent pregnancy in 12 of 15 German Shepherd bitches previously affected by prolonged secondary anoestrus. Because objective evidence of micronutrient or fatty-acid deficiencies was not obtained and no contemporaneous control group was available, causal inference is not possible. Nevertheless, the observations suggest that, in some breeding contexts, careful evaluation of dietary composition and nutrient bioavailability, including long-chain omega-3 fatty acids, arachidonic acid, folate, vitamin E and zinc, may warrant further study as potentially modifiable factors influencing cyclicity and conception. Clinicians evaluating infertile bitches may consider a focused dietary review and, where indicated, targeted nutrient assessment as part of the diagnostic workup. Nevertheless, prospective controlled studies incorporating baseline and follow-up nutrient status measurements and defined clinical outcomes are required before recommending routine use of multi-component supplements in the management of canine anoestrus.

## Limitations

This report has several important limitations that restrict interpretation of the findings. Baseline and follow-up measurements of circulating micronutrients, antioxidant status or fatty-acid profiles were not performed; consequently, the hypothesis that the affected bitches were nutritionally deficient remains unconfirmed. Individual daily food intake and total nutrient intake from the base diet were not directly measured, limiting precise estimation of nutrient exposure before and during supplementation. In addition, direct laboratory analysis of the commercial diet was not performed, and nutrient adequacy was therefore inferred from label declaration rather than measured composition. All animals received the same multi-component supplement, precluding identification of which individual nutrients, if any, might have influenced reproductive outcomes. The absence of a contemporaneous untreated or placebo group means that alternative explanations, such as spontaneous return to cyclicity, cannot be excluded. Although management conditions and housing remained unchanged throughout the observation period, and the preceding anoestrus had exceeded 12 months in all cases, these factors alone cannot fully exclude temporal or biological variability as contributors to the observed response.

## Data Availability

The raw data supporting the conclusions of this article will be made available by the authors, without undue reservation.
